# Spatiotemporal expression of SERPINE2 in the human placenta and its role in extravillous trophoblast migration and invasion

**DOI:** 10.1186/1477-7827-9-106

**Published:** 2011-08-02

**Authors:** Schu-Rern Chern, Sheng-Hsiang Li, Chien-Ling Chiu, Hsiao-Ho Chang, Chih-Ping Chen, Edmund I Tsuen Chen

**Affiliations:** 1 Department of Biotechnology and Laboratory Science in Medicine, National Yang-Ming University, Taipei, Taiwan; 2 Department of Medical Research, Mackay Memorial Hospital, Taipei, Taiwan; 3 Mackay Medicine, Nursing and Management College, Taipei, Taiwan; 4 Department of Obstetrics and Gynecology, Mackay Memorial Hospital, Taipei, Taiwan; 5 Department of Biotechnology, Asia University, Taichung, Taiwan; 6 School of Chinese Medicine, College of Chinese Medicine, China Medical University, Taichung, Taiwan; 7 Institute of Clinical and Community Health Nursing, National Yang-Ming University, Taipei, Taiwan; 8 Department of Obstetrics and Gynecology, School of Medicine, National Yang-Ming University, Taipei, Taiwan

## Abstract

**Background:**

SERPINE2, one of the potent serpins belonging to the plasminogen activator (PA) system, is involved in the tissue remodeling. We previously demonstrated the expression patterns of Serpine2 in the mouse placenta and uterus, indicating that Serpine2 is a major PA inhibitor in the placenta and uterus during the estrous cycle, pregnancy, and lactation. In this study, we further investigated the expression pattern of SERPINE2 in the human placenta and explored possible functional roles of SERPINE2 in regulating trophoblast activity.

**Methods:**

Placental tissues from various trimesters were collected for real-time reverse-transcription polymerase chain reaction quantification. Immunohistochemical staining was performed in placental tissues to assure localization of SERPINE2. *SERPINE2 *small interfering (si) RNA was applied to suppress its expression in villous explants and extravillous trophoblast-like 3A cells. Subsequent experiments to evaluate SERPINE2 levels, villous outgrowth, trophoblast invasion, and tube formation were performed.

**Results:**

*SERPINE2 *messenger RNA was detected in the human placenta during pregnancy with the highest levels in the third trimester. The SERPINE2 protein was present in villous syncytiotrophoblasts and trophoblasts of chorionic villi for anti-SERPINE2 immunostaining. Extravillous trophoblasts in the chorionic plate and basal plate confronting the invasive face of anchoring villi were also positive. In most decidual cells, SERPINE2 was observed in the cytoplasm. In addition, fibrinoid deposit was weakly immunoreactive. Introduction of *SERPINE2 *siRNA into villous explants and trophoblast cells led to significantly reduced villous outgrowth, and trophoblastic migration and invasion. Moreover, capillary-like network formation of 3A cells in Matrigel was greatly attenuated by *SERPINE2 *siRNA and SERPINE2 antiserum.

**Conclusions:**

These data identify the temporal and spatial SERPINE2 distribution in the human placenta and suggest its possible role in modulating tissue remodeling of extravillous trophoblasts in the placenta during pregnancy.

## Background

SERPINE2, also called protease nexin-1 and glial-derived neurite promoting factor, is a 44-kDa member of the serine protease inhibitor (SERPIN) superfamily. It was shown to be a potent inhibitor of the urokinase-plasminogen activator (uPA), tissue-type PA (tPA), thrombin, trypsin, factor XIa, and prostasin [1-5].

SERPINE2 is widely expressed in various tissues, including endothelial cells, fibroblasts, smooth muscle cells, tumor cells, glial cells, neurons, and placental cells [6-9]. Expression patterns of SERPINE2 in the placenta are quite dissimilar in different species. Expression levels of *SERPINE2 *in the monkey endometrium and placenta during early pregnancy were below the level of detection [10]. In rats, *Serpine2 *messenger RNA (mRNA) expression was only detected in endometrial stromal cells of the uterus, particularly at the time of implantation [11]. It was reported that SERPINE2 is highly expressed in the human placenta throughout pregnancy [12].

We demonstrated that Serpine2 is extensively expressed in various cell types in the mouse placenta and uterus, and in the human uterine endometrium [13,14]. In the murine uterus and placenta, it was prominently expressed in decidual stromal cells, metrial glands, endometrial epithelium, trophoblasts of the labyrinth, and spongiotrophoblasts during gestation. In humans, the SERPINE2 protein is highly expressed in the endometrium during the secretory phase [14]. These findings suggest a role for SERPINE2 in modulating tissue remodeling during implantation. Although SERPINE2 was found to be expressed by trophoblasts in various animals, the temporal expression of SERPINE2 in the human placenta during gestation still remains unclear [12].

Recent reports on human cancers indicated that SERPINE2 levels were elevated in pancreatic tumors [15], breast tumors [16], colorectal tumors [17], oral squamous carcinomas [18], and liposarcomas [19]. In contrast, the physiological function of SERPINE2 in placental extravillous trophoblasts that possess "pseudomalignant" features is less well documented [20].

In addition to previous findings of the relatively abundant levels of SERPINE2 in female reproductive tissues, existing microarray gene expression profiles of normal human tissues deposited in the NCBI GEO database (http://www.ncbi.nlm.nih.gov/geo/; GDS596, GDS1096, and GDS3113) show that the placenta expresses the highest levels of *SERPINE2 *among all probed tissues except seminal vesicles. In the present study, we investigated the spatiotemporal expression of SERPINE2 in the human placenta. Further, knock-down experiments with *SERPINE2 *were performed to examine if the suppression of *SERPINE2 *in villous explants and trophoblast cells could modulate trophoblast invasion *in vitro*.

## Methods

### Placental tissue collection

Human placental tissues from the first (7~12 wk of gestation; *n *= 5), second (13~24 wk of gestation; *n *= 4), and third trimesters (31~38 wk of gestation; *n *= 10) were obtained from the Department of Obstetrics and Gynecology, Mackay Memorial Hospital. Signed, written consent was obtained from each patient before sample collection. The use of placental tissue specimens and the consent forms were approved by the Institutional Review Board of Mackay Memorial Hospital. Tissues were collected and washed three times in sterile saline, then they were (a) fixed in 10% neutral formalin (Merck, Darmstadt, Germany), embedded in paraffin, (b) stored in either RNAlater (Ambion, Austin, TX, USA) at -80°C for subsequent RNA extraction, and/or (c) finely minced with a surgical knife and resuspended in culture medium (Medium 199 containing 10% fetal calf serum (FCS), penicillin/streptomycin, and amphoteracin B).

### Cell/explant culture and treatment

3A cells, derived from first-trimester human trophoblast by SV40 ts30 transformation [21], were purchased from ATCC (CRL-1584; Rockville, MD, USA). Cells were cultured in medium 199 (M199, Invitrogen, Carlsbad, CA, USA) supplemented with 10% FCS (Invitrogen) and 100 IU/ml penicillin/streptomycin (Invitrogen), and maintained at 37°C in 5% CO_2_. Preparation of placental explants was performed as described elsewhere [22]. Villous tips were dissected under a microscope to approximately 2~3 mm^3 ^and incubated for 16 h in culture medium at 37°C and 5% CO_2_. An extracellular matrix (ECM) gel solution was prepared using 1 ml neutralized collagen I (4 mg/ml, Sigma-Aldrich, St Louis, MO, USA) mixed with 1 ml Matrigel (10 mg/ml, BD Biosciences, San Jose, CA, USA). The ECM gel for explants was formed by adding 85 μl of the ECM solution onto the surface of 6-well culture dishes (Nunc, Roskilde, Denmark). After hardening of the gels, the villous tips (explants) were put on top of the gel and immersed in medium for 2~4 h to allow adherence to the ECM. Explants were then flooded with 1 ml M199 medium in the absence or presence of small interfering RNA (siRNA). Three ECM gels in each well containing 9~12 and 30~36 explants of each placenta were utilized. Four placentas (with respective gestational ages of 9, 12, 16, and 20 wk) were subjected to explant culture. Villous outgrowth and migration of trophoblasts were evaluated following a previously published protocol [23]. Briefly, the explant morphology was carefully analyzed by two observers to judge explant outgrowth. The numbers of outgrowth sites of villous explants were recorded at 72 h. Migration of trophoblast cells in the ECM was scored at 120 h on a scale of 0-5 (0, no migration; 1, one or two sites of localized migration; 2, several sites of localized migration; 3, moderate migration; 4, moderate to extensive migration; and 5, extensive migration from several sites around the explant).

*SERPINE2*-targeted siRNAs and corresponding scrambled siRNAs were obtained from Sigma-Aldrich (for sequences see Additional file 1, Table S1). 3A cells (3 × 10^6^) were transiently transfected with 0.05 μM of siRNAs using the Lipofectamine 2000 (Invitrogen) reagent, whereas villous cultures directly gained 1 μM siRNAs by an active transport mode [24]. After 24 h of transfection, 3A cells were trypsinized for subsequent experiments, including RNA extraction following a reverse-transcription polymerase chain reaction (RT-PCR), wound-healing assay, invasion assay, and tube formation as described below. During the culture of explants, the medium was collected at 16 and 24 h for the Western blot analysis. At the end of the experiment, explants were harvested and processed for Western blotting, RNA extraction, and RT-PCR.

A batch of explants (at a gestational age of 14 wk) was cultured for 10 days in Matrigel to allow the ECM gel shank. The gel containing villi was fixed in 10% neutral buffered formalin for 24 h and processed for the immunohistochemical analysis.

### Immunocytochemistry

Immunocytochemical staining was performed as previously described [14]. Briefly, placental tissues were collected, fixed in neutralized formalin, embedded in paraffin, and cut into 5-μm sections. After the sections were deparaffinized and hydrated, they were treated with 3% hydrogen peroxide in phosphate-buffered saline (PBS, Invitrogen) for 15 min to quench any endogenous peroxidase activity, then blocked with 10% normal goat serum in PBS for 1 h. Immunocytochemistry for the SERPINE2 and trophoblast-specific CK7 marker was performed on tissues using anti-SERPINE2 antiserum (1:700) [14] and anti-human CK7 (1:100) (Dako, Copenhagen, Denmark) in blocking solution at 4°C for 16 h. Negative controls were performed by replacing primary antibodies with serum pretreated with SERPINE2-conjugated beads at the same concentration. After washing, slides were treated with biotin-conjugated goat anti-rabbit immunoglobulin G (IgG; ~3 μg/ml) (Zymed, San Francisco, CA, USA). Slides were then incubated with horseradish peroxidase (HRP)-conjugated streptavidin (~1 μg/ml) (Zymed) in the blocking solution for 40 min at room temperature. Protein signals were detected by 3-amino-9-ethylcarbazole (Zymed) staining. Slides were then counterstained with hematoxylin (Vector Laboratories, Burlingame, CA, USA) and photographed using a Zeiss AxioImager Z1 microscope system (Wetzlar, Germany) equipped with a digital camera and an automated acquisition system (TissueGnostics, Vienna, Austria).

### RNA isolation and real-time RT-PCR

Total RNA of placental tissues and 3A cells was extracted using a QuickPrep RNA extraction kit (GE Healthcare Life Sciences, Uppsala, Sweden). To examine *SERPINE2 *gene expression, an RT-PCR was conducted. PCR primers (for sequences see Additional file 1, Table S1) were designed to cross the junction between the exon and intron. Four micrograms of total RNA was reverse-transcribed using an iScript complementary DNA synthesis kit (Bio-Rad, Hercules, CA, USA) according to the manufacturer's instructions. The PCR was performed in a total volume of 20 μl, and 25 ng of cDNA was added to the PCR mix made with 2-fold pre-mix containing fluorescein (Kapa SYBR Fast qPCR Kit, KAPA Biosystems, Woburn, MA, USA). Amplification conditions were as follows: 95°C for 10 min, and then 40 cycles at 95°C for 15 s and at 60°C for 1 min using the CFX96 real-time PCR system (BioRad) in triplicate. The PCR amplification efficiency for each gene was tested to be sure it was equivalent to that of *RPLPO mRNA *examined in a cDNA dilution series. Product purity was checked through a melting curve analysis at the end of the real-time PCR. The accuracy of the PCR products was confirmed by DNA sequencing. Relative gene expression levels were determined using the threshold cycle (CT) method (2^-ΔΔCt ^method) with reference to the endogenous *RPLPO *control [25]. Gene expression was normalized to RNA loading using primers for *RPLPO *as an internal standard.

### Western blot analysis

Tissue extract proteins were separated by sodium dodecylsulfate-polyacrylamide gel electrophoresis (SDS-PAGE) on a 4%~15% gradient gel (Invitrogen) and were transferred to nitrocellulose membranes for immunostaining. Membranes were blocked with 10% (w/v) skim milk in PBS (blocking solution) for 2 h, and then incubated with anti-SERPINE2 antiserum (1:3000) or monoclonal anti-β-actin (1:5000, Sigma-Aldrich) in blocking solution for 1 h at room temperature. After gentle agitation in four changes of PBS for 15 min each, the membranes were immunoreacted with HRP-conjugated goat anti-rabbit IgG (1:10000, GE Healthcare Life Sciences) or HRP-conjugated anti-mouse IgG (1:15000; GE Healthcare Life Sciences) in blocking solution for 1 h. Immunoreactive bands were revealed using an enhanced chemiluminescence (ECL) substrate according to the manufacturer's instructions (Pierce, Rockford, IL, USA).

Collected medium (25 μl) from explants and 3A cell cultures were subjected to Blue Sepharose (GE Healthcare Life Sciences) chromatography to remove albumin and analyzed by Western blotting. Residual albumin was taken as the loading control and immunodetected by a rabbit anti-BSA polyclonal antibody (1:2500, GeneTex, San Antonio, TX, USA).

### Invasion assay

Invasion assays were carried out using Matrigel Invasion Chambers (BD Biosciences). Briefly, cells transfected with siRNAs (scrambled or *SERPINE2*) were trypsinized and seeded on Matrigel inserts in triplicate at a density of 5 × 10^4 ^cells/well in 200 μl of culture medium. Following an incubation period of 48 h, the filter inserts were removed and washed three times with PBS. Cells were fixed with 4% paraformaldehyde (Merck) and stained with hematoxylin for 3 min. Cells on the upper side of the transwell membrane were removed with a cotton tip swab. Cell numbers on the filters were counted under a microscope (IX71, Olympus, Tokyo, Japan).

### Migration scratch assay

3A cells with or without siRNA treatment were harvested after 24 h of transfection, seeded into 10-cm dishes, and grown to about 95% confluence. A wound about 1 mm wide was created on cell monolayers by scraping them with a pipette tip. The dishes were washed with PBS to remove detached cells and incubated for 16 h at 37°C in the culture medium. After incubation, images were taken on an inverted light microscope (Olympus IX71) equipped with a digital camera. For quantification, these images were analyzed with the TScratch software tool [26]. This free software is able to automatically measure the area occupied by cells in the image. The cell-free area correlated with the ability of 3A cells to migrate into the scratch, and closure of the controls was used for normalization.

### In vitro angiogenesis assay (tube-formation assay)

Matrigel was thawed on ice overnight, and 10 μl was pipetted with ice-cold pipette tips into the lower chambers of an angiogenesis slide (IBIDI, München, Germany) and allowed to harden for 30 min at 37°C. Then, 7500 cells with or without siRNA treatment were seeded on the chamber and incubated overnight in the absence or presence SERPINE2 anti-serum (1:100) in the medium. Images were taken using an Olympus DP71 microscope with a camera system and analyzed using Image-Pro Plus software vers. 6.0 (Media Cybernetics, Houston, TX, USA). Network formation was quantified by measuring the branch point, and total cord lengths were compared between silenced and non-silenced cells.

### Statistical analysis

Data are expressed as the mean ± standard error of the mean (SEM). Results were subjected to a statistical analysis by Student's *t*-tests as appropriate using Prism 5.0c (GraphPad Prism, San Diego, CA, USA), and significance was accepted when *p *< 0.05. * *p *< 0.05; ** *p *< 0.01; *** *p *< 0.001. All experiments were repeated three or more times with similar results.

## Results

### Spatiotemporal expression of SERPINE2 in the human placenta

**Figure 1:**
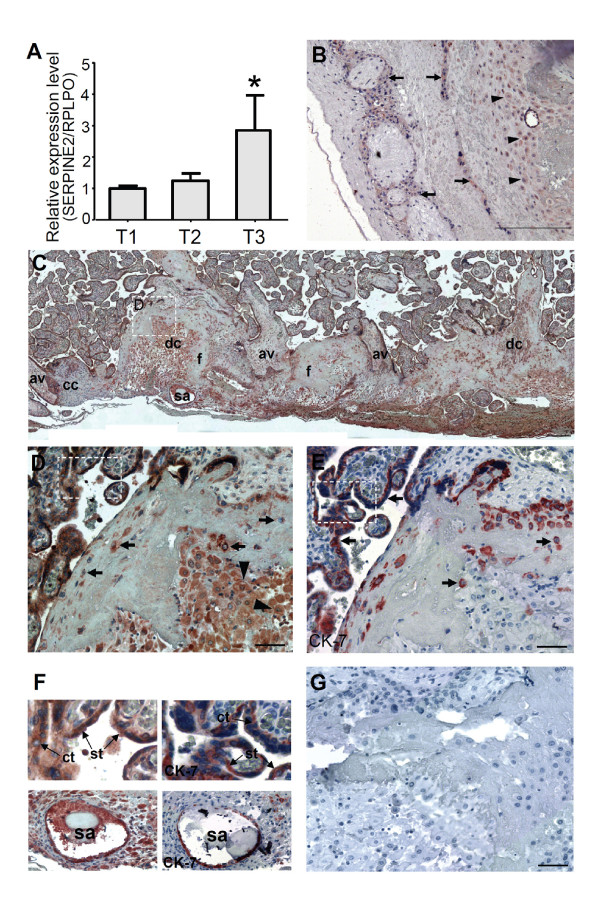
**Spatiotemporal expression of SERPINE2 in the human placenta**. (A) Expression of *SERPINE2 *mRNA in human placentas in different trimesters. Levels of *SERPINE2 *mRNA were determined by real-time quantitative PCR in 19 placental tissues collected in three gestational trimesters (T1, T2, and T3; *n *= 5, 4, and 10, respectively). For each sample, the *SERPINE2 *expression level was normalized to the expression level of the *RPLPO *gene in the same sample. Data are presented as the mean ± SEM. * *p *< 0.05. Immunohistochemical staining showed the distribution of the SERPINE2 protein in the human placenta at gestational week 15. (B) Moderate staining of extravillous trophoblasts (arrow) and decidual cells (arrowhead) in the chorionic plate, and very low staining in the chorionic mesoderm and fibrinoid deposits. (C) Positive immunostaining was extensively detected in decidual cells (dc), cytotrophoblasts, extravillous trophoblasts at the junction zone of the cell column (cc) and anchoring villi (av), and the endothelia of the spiral artery (sa); and weak staining was found in fibrinoids (f) and the villous mesenchyme. (D) The dashed-lined region in 1C was magnified to show intense staining in syncytiotrophoblasts and cytotrophoblasts in floating villi. The invaded extravillous trophoblasts (arrow) and decidual cells (arrowhead) at the basal plate were strongly stained. (E) Positive staining of cytokeratin (CK)-7 was confirmed in syncytiotrophoblasts and cytotrophoblasts in floating villi, and invading trophoblasts (arrow). (F) Upper panels, dashed-lined rectangle regions in D and E were magnified to show strong staining of SERPINE2 (left) and CK-7 (right) in syncytiotrophoblasts (st) and cytotrophoblasts (ct) in floating villi. Lower panels, most of the endothelia of spiral arteries were positively stained with anti-SERPINE2 (left), and anti-CK-7 (right) antibodies. (G) Negative staining of control antiserum. Scale bars represent 200 μm (B, C), and 50 μm (D, E, G).

We analyzed the expression of *SERPINE2 *in human placentas from various trimesters (*n *= 5, 4, and 10 for the first, second, and third trimesters, respectively) at the mRNA level by real-time RT-PCR. The level of *SERPINE2 *mRNA was about 2.7-fold higher in the third trimester compared to the first trimester (Figure 1A).

Immunohistochemistry was used to localize SERPINE2 in placental tissues obtained from the second trimester (*n *= 3). SERPINE2 protein expression was visible in extravillous trophoblast cells and decidual cells of the chorionic plate (Figure 1B). However, it was strongly expressed in villous syncytiotrophoblasts, cytotrophoblasts, decidual cells, extravillous trophoblasts anchoring the villous cell column (av-cc) adjunction zone, spiral arteries, and decidual stroma cells (Figure 1C, D). The immunoreaction was evident in extravillous trophoblasts invading the basal plate, where cytoplasm of most decidual cells were positively stained (Figure 1D). No detectable signal was found in septal endometrial stromal cells or mesenchymal cores of chorionic villi. Lower immunoreactivity was observed in fibrinoid deposits (Figure 1D). CK-7-positive extravillous trophoblasts were observed in the cell column (Figure 1E). Syncytiotrophoblasts and cytotrophoblasts in floating villi were strongly positive for SERPINE2 (Figure 1F, upper-left panel). Furthermore, most endothelia of spiral artery were both SERPINE2 and CK-7 positive (Figure 1F, lower panels). However, when slides were immunostained with control antiserum, no signal was detected (Figure 1G).

### Villous explants

**Figure 2:**
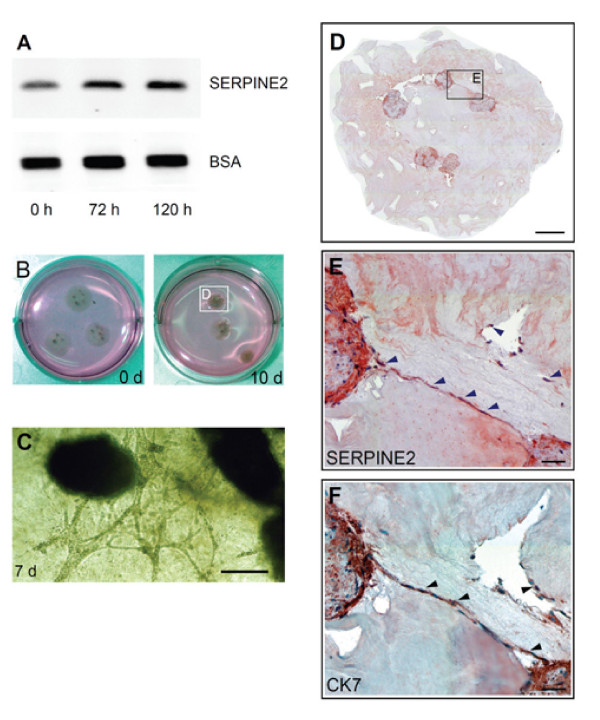
**Trophoblast outgrowth and invasion of villous explants on the extracellular matrix (ECM) gel accompanying elevated SERPINE2 expression**. (A) Western blot analysis was used to detect the secreted SERPINE2 protein in the supernatant of explant cultures. Significant increases of the explants during culture were evidenced compared to the loading controls (BSA from FBS in the culture medium, partially removed by affinity chromatography). (B) After long-term (10-day) explant culture, the gels had shrunk and formed condensed 'balls' and probably detached from the plastic surface of the culture dish to float in the medium. (C) Before condensation of the ECM gel, a 3D mesh could be observed under an inverted microscope. An illustrative photo of a 7-day explant is shown. (D) Explants in shrunken gel were harvested and immunochemically stained with SERPINE2 antiserum. (E) The invading, lined trophoblasts were immunoreactive with SERPINE2 (arrowhead). (F) The invading cells were mostly CK-7-positive trophoblasts. Scale bars represent 500 μm (C, D), and 100 μm (E, F).

Villous explant culture on Matrigel was demonstrated as an *in vitro *model that allows the study of extravillous trophoblast outgrowth, migration, and invasion during the first trimester of gestation [22]. We preliminarily found that more SERPINE2 proteins were secreted into the culture medium during the outgrowth of explant villi and the invasion of trophoblasts (Figure 2A). Villi embodied within the shrunken gel (Figure 2B) at 10 d were examined with an immunohistochemical analysis. Trophoblasts with SERPINE2 staining had migrated, invaded, and formed networks among the gels (Figure 2C-F).

The trophoblast-derived cell line, 3A-sub E (3A) [21,27], was applied for the *in vitro *studies of SERPINE2 in extravillous trophoblasts. Of note, 3A cells express HLA-G, CD9, and CK-7 mRNAs, which are characteristics of invasive extravillous trophoblasts [27,28], as shown by an RT-PCR study (Additional file 2, Figure S1). Furthermore, *SERPINE2 *mRNA levels in 3A cells resembled those of the first trimester placenta tissue (data not shown).

### Knockdown of *SERPINE2*

**Figure 3:**
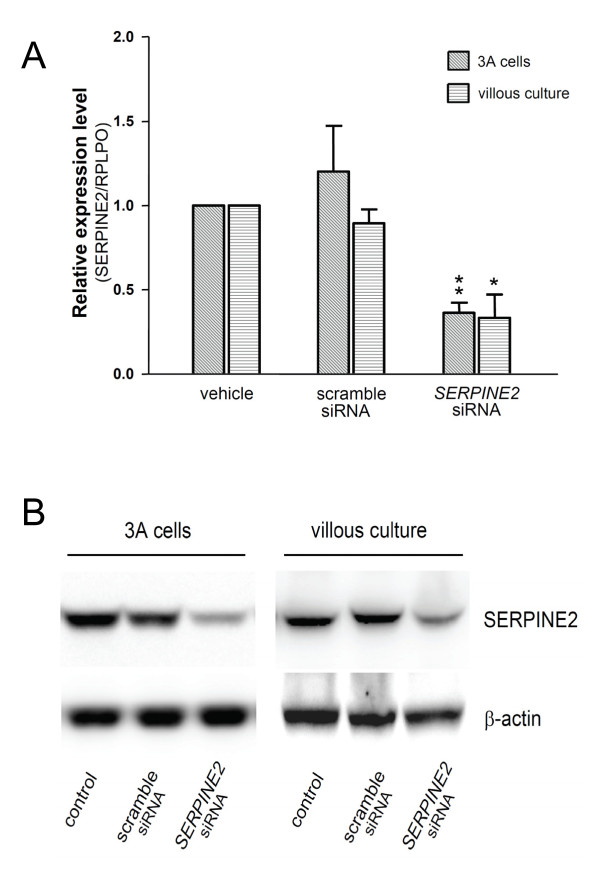
***SERPINE2 *siRNA decreases expressions of *SERPINE2 *mRNA and protein in 3A cells and villous culture**. (A) *SERPINE2 *mRNA levels in 3A cells and villous culture were specifically blocked by siRNA against *SERPINE2*. Scrambled or *SERPINE2 *siRNAs were delivered into cells by transfection (3A cells) or by active transport (villous culture). *SERPINE2 *mRNA was determined by real-time quantitative PCR after 48 h of treatment. Data are presented as the mean ± SEM. * *p *< 0.05, ** *p *< 0.01 were considered significant. (B) Western blot analysis of cell lysates isolated from siRNA-treated (48 h) villi and 3A cells. For the loading control, membranes were stripped and re-incubated with a β-actin antibody.

**Figure 4:**
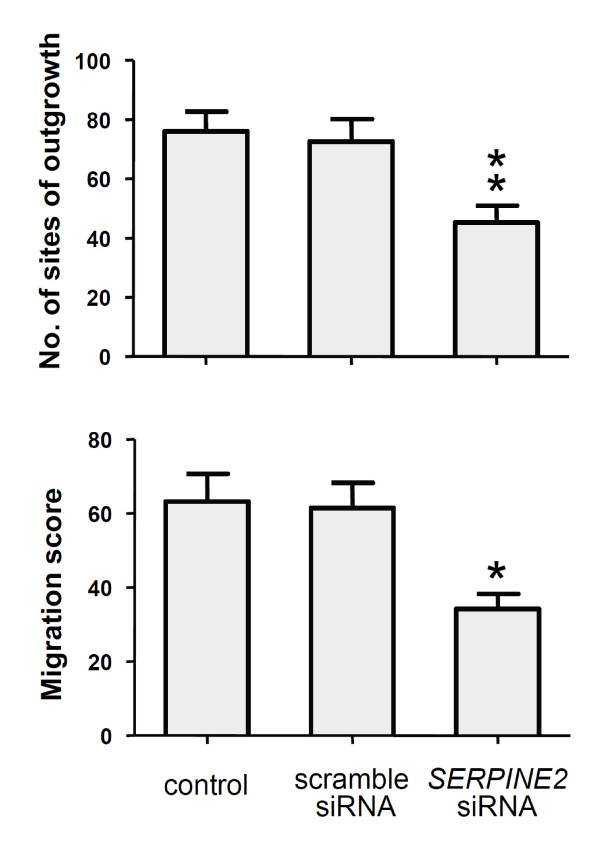
**Semiquantitative analysis of villous explant outgrowth and migration**. Explants from four placentas (at the gestational ages of 9, 12, 16, and 20 wk) were cultured in the presence or absence of *SERPINE2 *siRNA for 120 h, and the migration score based on a scale (see Methods and Additional file 3, Figure S2) was evaluated by 2 observers. Data are presented as the mean ± SEM. * *p *< 0.05 ** *p *< 0.01.

**Figure 5:**
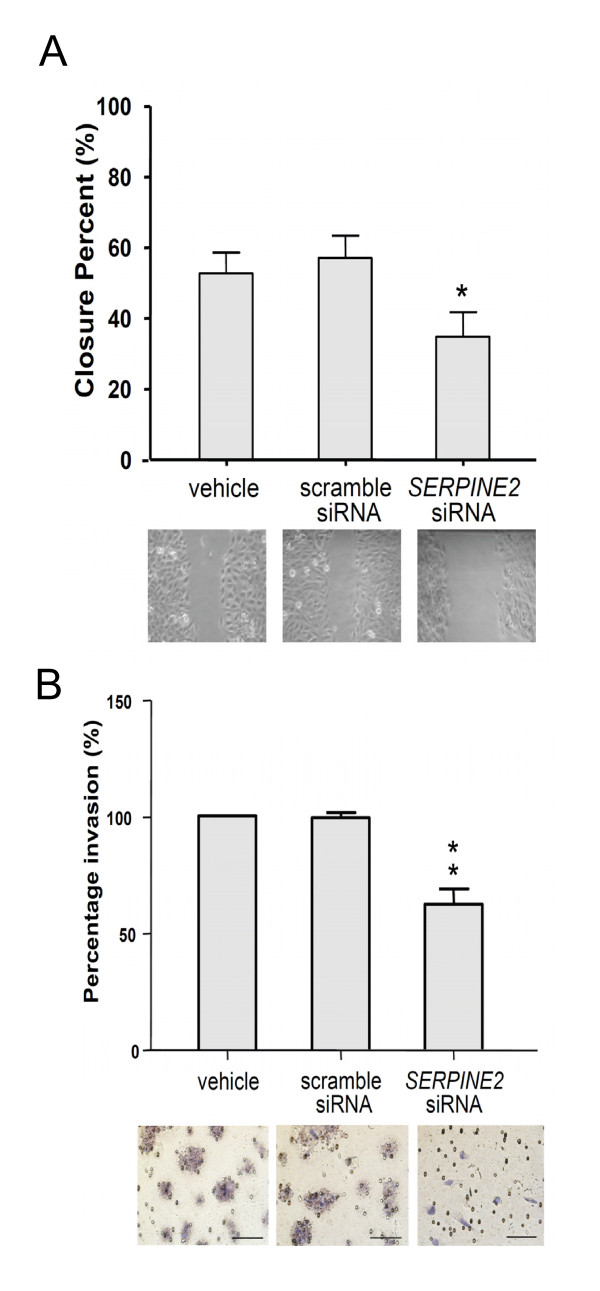
**Suppression of *SERPINE2 *led to inhibition of the migration and invasion of trophoblast-derived 3A cells**. (A) A motility assay demonstrated deduced cell migration of 3A cells transfected with siRNA which specifically silenced the expression of *SERPINE2*. (B) A Matrigel invasion assay showed a reduced invasive capacity of 3A cells treated with *SERPINE2 *siRNA. Percentage invasion of vehicle is set as 100%. * *p *< 0.5, ** *p *< 0.01. Data are presented as the mean ± SEM. Scale bars represent 100 μm.

In our examination of the effect of siRNA-mediated downregulation of *SERPINE2 *(Figure 3), both mRNA and protein levels of *ex vivo *villous cultures and 3A cells were specifically suppressed. A scoring scale is illustrated (Additional file 3, Figure S2) to show explant outgrowth and invasion of trophoblasts. The depletion of SERPINE2 substantially suppressed villous outgrowth and the invasion of trophoblasts in ECM gels (Figure 4). Cells deprived of *SERPINE2 *exhibited reduced migration and invasion (Figure 5). Wound-healing assays demonstrated that *SERPINE2 *siRNA reduced the motility of 3A cells on plastic (Figure 5A). Compared to the controls, *SERPINE2*-silenced cells were significantly less migratory (52.7% ± 5.9% vs. 34.8% ± 7%, *p *< 0.01). Likewise, siRNA reduced the invasion of 3A cells through Matrigel-coated transwells (Figure 5B). The percentage of cells reaching the undersurface of the membrane had decreased to 70.4% (*p *< 0.01) after 48 h. To exclude that the effects of siRNA on invasion/motility were caused by a decrease in the proliferative capacity of cells, accumulative cell numbers were determined (Additional file 4, Figure S3).

### Tube-formation assay

**Figure 6:**
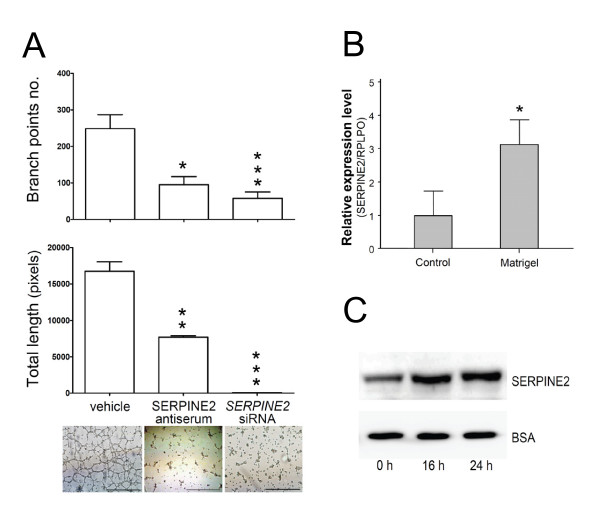
**Matrigel-induced network formation of trophoblast 3A cells was inhibited by SERPINE2 antiserum or siRNA**. (A) Treatment of 3A cells with SERPINE2 antiserum or siRNA significantly blocked the formation of the capillary-like network, as scored by the number of branch points and total length formed following 24 h of treatment. (B) *SERPINE2 *mRNA levels in 3A cells in Matrigel compared to controls. (C) Western blot analysis was used to detect the secreted SERPINE2 protein in the supernatant of 3A cells in the tube formation assay. Each experiment was carried out in triplicate, and each bar represents the mean ± SEM. * *p *< 0.05, ** *p *< 0.01, *** *p *< 0.001 compared to the scrambled siRNA-treated controls. Representative images of capillary-like network formation by 3A cells are shown. Scale bars represent 100 μm.

Extravillous trophoblast cells were found to develop endothelial cell-like behavior when cultivated on a matrix such as Matrigel. They migrated in the matrix to form networks of tube-like structures [29,30]. Similar phenomena were obtained from cultures of 3A cells on Matrigel (Figure 6A, lower panel). mRNA levels of *SERPINE2 *in 3A cells cultured on Matrigel were promoted to about 3.1-fold compared to cells cultured on plastic as control (Figure 6B). Secreted SERPINE2 was analyzed and found elevated during capillary formation (Figure 6C). We used 3A cells and an angiogenesis slide coated with Matrigel to investigate the effect of inhibiting SERPINE2 on cell network formation. Compared to the controls, the number of networks and total length of capillaries were significantly reduced in the subset treated with *SERPINE2 *siRNA and in the subset incubated with SERPINE2 anti-serum (Figure 6A, upper panel). Immunofluorescent staining was also performed to assure the localization of SERPINE2 in the formed capillary tubes (Additional file 5, Figure S4).

## Discussion

In this study, we demonstrated that SERPINE2 was extensively expressed in several cell types that exist in the human placenta. It was highly expressed in syncytiotrophoblasts, cytotrophoblasts, and extravillous trophoblasts in the placenta, while it was relatively weakly expressed in decidual cells (Figure 1D). These results were similar to a previous report on the term human placentas [12] which showed strong SERPINE2 immunofluorescence in amnion, chorion, and trophoblastic epithelia; lower intense and interspersed signals in decidual cells and stroma. We further specified the co-localization of SERPINE2 and invaded extravillous trophoblasts at the basal plate and the endothelium of the spiral artery. Recently, we demonstrated that Serpine2 was extensively expressed in labyrinthine trophoblasts, spongiotrophoblasts, and decidual cells, but was less expressed in giant cells in the mouse placenta. The expression pattern of the placental SERPINE2 protein greatly differs in various species. In rhesus monkeys, SERPINE2 protein expression in the compartments of the endometrium and placenta was lower or undetectable [10]. However, the SERPINE2 protein is extensively expressed in human and murine placentas.

Interestingly, the pattern of SERPINE2 expression in the human placenta presented here during gestation parallels circulating uPA and tPA levels during pregnancy [31,32]. However, SERPINE2 has broad-spectrum activity specific to serine proteases, including trypsin, thrombin, factor XIa [4], and prostasin [5]. The question of what the cognate protease is in the human placenta needs to be further investigated.

Serine proteases and their cognate inhibitors were documented to play key roles in matrix remodeling and degradation in the uterus. Accordingly, a well-regulated balance between levels of protease and its inhibitor during endometrial matrix remodeling and restricted trophoblast invasion are essential for a successful pregnancy [33]. We herein showed that downregulation of *SERPINE2 *mediated by siRNA effectively suppressed the outgrowth of villous explants and tended to inhibit extravillous trophoblast invasion (Figure 4). In trophoblast-derived 3A cells, *SERPINE2 *siRNA significantly impaired both migration and invasion (Figure 5). It seems that the depletion of SERPINE2 disturbs the balance between proteases and protease inhibitors, causes a shift in the ECM profile, and further affects the activity of trophoblasts.

During the first trimester of pregnancy, spiral arteries transform into wide-caliber, low-resistance vessels, extravillous trophoblasts invade through the maternal decidua to the spiral arteries, and then differentiate into tube structures, lining the artery walls with endothelial cells. Blockage of SERPINE2 inhibited 3A cell formation of capillary-like networks in Matrigel (Figure 6B), suggesting that SEPINE2 is essential for invading trophoblasts to develop vasculogenic mimicry. We therefore propose the following scenario for the regulation of extravillous trophoblasts in tissue remodeling by SERPINE2. SERPINE2 in the ECM establishes a micro-environment that facilitates trophoblast cell migration, and further promotes trophoblast cells aligning with themselves or with the maternal endothelium to establish vasculature structures via an unknown signaling pathway. Additionally, SERPINE2 in the ECM may originate from trophoblasts themselves or from maternal decidual cells in a coordinated manner.

Plasminogen activator inhibitor-1 (PAI-1 or SERPINE1), the phylogenetically closest relative of SERPINE2, is more deeply studied, as exemplified by a body of published work [34]. Actually, *SERPINE1 *mRNA seems to be the major PA inhibitor in the human placenta (our unpublished data). The uPA-uPAR system and PAI-1 are undoubtedly associated with malignances through regulating tumor cell proliferation, migration, invasion, metastasis, and apoptosis [35-38], although contrary to what would be expected for the function of a cellular protease inhibitor. Likewise, SERPINE2 is reported to be upregulated in various tumors [15-19]. Further studies of serine proteases and cognate inhibitors' effects on placental tissue development will greatly enhance our understanding of the physiological and pathophysiological mechanisms of tissue remodeling.

## Conclusions

*SERPINE2 *is highly expressed in the human placenta during pregnancy. Blockage of SERPINE2 results in inhibition of extravillous trophoblast activity *in vitro*, including migration, invasion, and tube formation. Taken together, these data suggest that SERPINE2 plays important roles in modulating placental tissue remodeling during pregnancy. Our studies may provide helpful insights into the roles of SERPINE2 in placental remodeling.

## Competing interests

The authors declare that they have no competing interests.

## Authors' contributions

SRC carried out Western blotting, evaluated the activity of villous trophoblasts, and drafted the manuscript. SHL helped draft the manuscript. CLC carried out real-time PCR analyses and cell culture. HHC carried out immunohistochemistry. CPC and EITC conceived the study, and participated in the project design and coordination. All authors read and approved the final manuscript.

## Supplementary Material

Additional file 1 **Supplemental Table S1: Sequences of real-time PCR primers and siRNAs**. Click here for file

Additional file 2 **Supplemental figure S1: Characterization of the trophoblast 3A cell line by RT-PCR analysis**. Click here for file

Additional file 3 **Supplemental figure S2: Illustrated pictures of scoring scale of the villous explant migration base on a ordered series**. Click here for file

Additional file 4 **Supplemental figure S3: Proliferation assay using an Alamar Blue dye reduction analysis of the viability of 3A cells after siRNA treatment**. Click here for file

Additional file 5 **Supplemental figure S4: Immunofluorescence analysis of the network formed in the tube-formation (micro-angiogenesis) assay**. Click here for file
