# Supplementary material for: Spatiotemporal expression of SERPINE2 in the human placenta and its role in extravillous trophoblast migration and invasion
Source: Reprod Biol Endocrinol. 2011 Aug 2;9:106. doi: 10.1186/1477-7827-9-106 (PMC3161939; doi:10.1186/1477-7827-9-106)
Supplement: Additional file 1 — Supplemental Table S1: Sequences of real-time PCR primers and siRNAs. [file 1477-7827-9-106-S1.DOC]

Table S1 Sequences of real-time PCR primers and siRNAs

| Gene ^a^ | Sequence (5’ → 3’) | Position (nt) | Product size (bp) |
| --- | --- | --- | --- |
| *SERPINE2* | | | |
|  | Forward: AGATGTGTTCCAGTGTGAGGTCC | 636~658 | 222 |
|  | Reverse: CGTTTCTTTGTGTTCTCGGGTT | 857~836 |  |
| *RPLPO* | | | |
|  | Forward: CAACCCTGAAGTGCTTGATAT | 774~794 | 227 |
|  | Reverse: AGGCAGATGGATCAGCCA | 1000~983 |  |
| *SERPINE2* siRNA | | | |
|  | Sense: CAGUGUAUUUCAAGGGUCUUU | 800~818 |  |
| Scrambled siRNA | | | |
|  | Sense: FAM-UUCUCCGAACGUGUCACGUTT | - |  |

^a^ GenBank accession nos.: *SERPINE2*, NM_006216.3; *RPLPO*, NM_001002.3.
