# Supplementary material for: Spatiotemporal expression of SERPINE2 in the human placenta and its role in extravillous trophoblast migration and invasion
Source: Reprod Biol Endocrinol. 2011 Aug 2;9:106. doi: 10.1186/1477-7827-9-106 (PMC3161939; doi:10.1186/1477-7827-9-106)
Supplement: Additional file 2 — Supplemental figure S1: Characterization of the trophoblast 3A cell line by RT-PCR analysis. [file 1477-7827-9-106-S2.PDF]

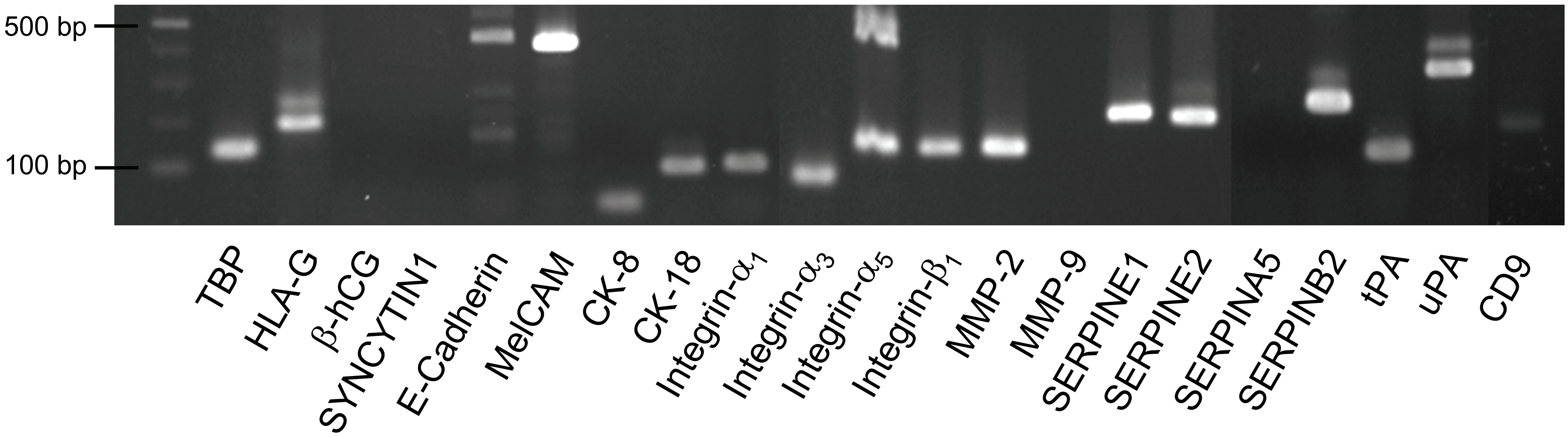

Figure S1 Characterization of the trophoblast 3A cell line by RT-PCR analysis. cDNA (20 ng) was applied in each test, and a thermal program (40 cycles of 95 ˚C for 30s, 57 ˚C for 20s, and 70 ˚C for 30s) was performed to detect mRNAs of various genes. PCR products were resolved by gel electrophoresis: lane 1, molecular weight standard; lanes 2~20 were TBP (internal control), HLA-G, β-hCG, SYNCYTIN1, E-cadherin, MelCAM, CK8, CK18, Integrin α 1, Integrin α 3, Integrin α 5, Integrin β 1, MMP-2, MMP-9, SERPINE1, SERPINE2, SERPINA5, SERPINB2, tPA, uPA, and CD9.
