# Supplementary material for: Spatiotemporal expression of SERPINE2 in the human placenta and its role in extravillous trophoblast migration and invasion
Source: Reprod Biol Endocrinol. 2011 Aug 2;9:106. doi: 10.1186/1477-7827-9-106 (PMC3161939; doi:10.1186/1477-7827-9-106)
Supplement: Additional file 3 — Supplemental figure S2: Illustrated pictures of scoring scale of the villous explant migration base on a ordered series. [file 1477-7827-9-106-S3.PDF]

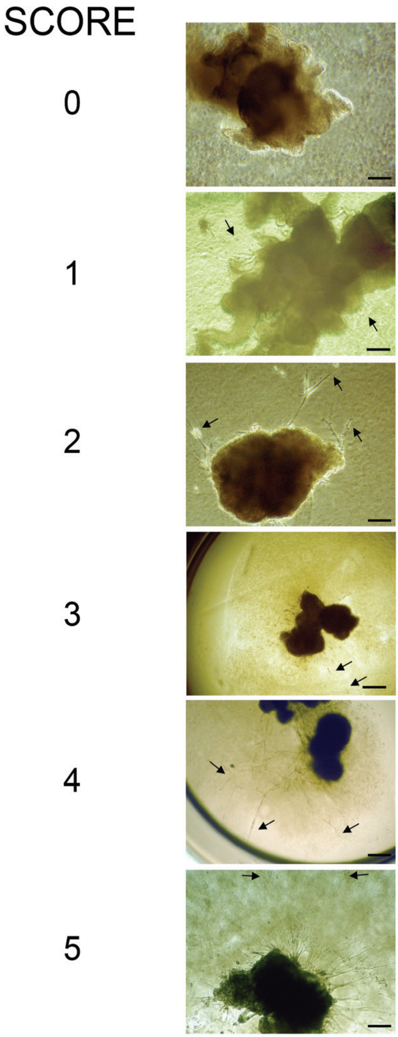

Figure S2 Illustrated pictures of scoring scale of the villous explant migration base on a ordered series: 0, no migration; 1, 1~2 sites of localized migration; 2, more than 3 sites of localized migration; 3, moderate migration; 4, moderate to extensive migration; and 5, extensive migration from several sites around the explant [23]. Arrows indicate invaded trophoblast. Scale bars represent 100 μm .
