# Supplementary material for: Spatiotemporal expression of SERPINE2 in the human placenta and its role in extravillous trophoblast migration and invasion
Source: Reprod Biol Endocrinol. 2011 Aug 2;9:106. doi: 10.1186/1477-7827-9-106 (PMC3161939; doi:10.1186/1477-7827-9-106)
Supplement: Additional file 4 — Supplemental figure S3: Proliferation assay using an Alamar Blue dye reduction analysis of the viability of 3A cells after siRNA treatment. [file 1477-7827-9-106-S4.PDF]

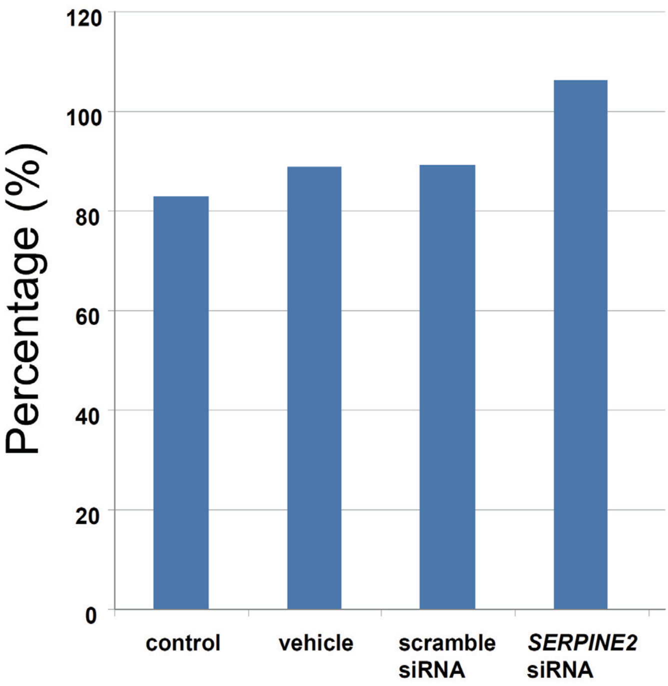

Figure S3 Proliferation assay using an Alamar Blue dye reduction analysis of the viability of 3A cells after siRNA treatment. 3A cells (3 x 105) were seeded on 6-well plates and incubated overnight, then transfected with siRNAs; the vehicle wells contained only transfectant. After 24 h, Alamar Blue (AB) dye (Invitrogen) was added to the medium, and the percent AB reduction was measured after another 24 h. The percentage of proliferation was then deduced by normalization to a previously established standard curve.
