# Supplementary material for: Spatiotemporal expression of SERPINE2 in the human placenta and its role in extravillous trophoblast migration and invasion
Source: Reprod Biol Endocrinol. 2011 Aug 2;9:106. doi: 10.1186/1477-7827-9-106 (PMC3161939; doi:10.1186/1477-7827-9-106)
Supplement: Additional file 5 — Supplemental figure S4: Immunofluorescence analysis of the network formed in the tube-formation (micro-angiogenesis) assay. [file 1477-7827-9-106-S5.PDF]

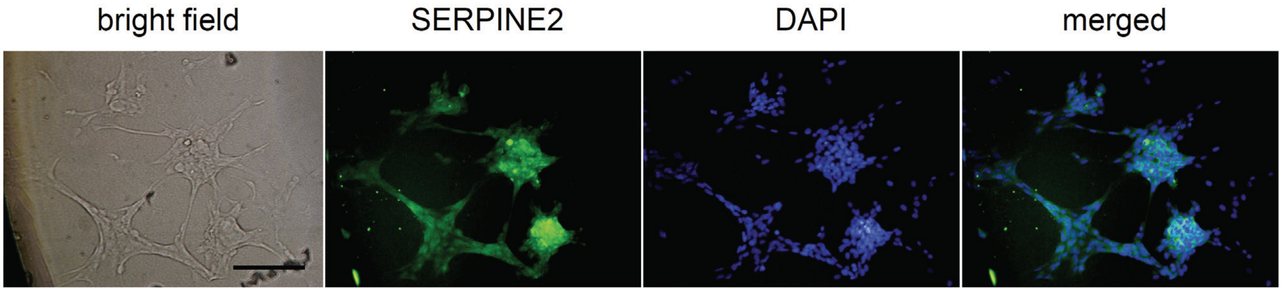

Figure S4 Immunofluorescence analysis of the network formed in the tube-formation (micro-angiogenesis) assay. 3A cells were grown in Matrigel-coated micro-angiogenesis wells, formed a network at 24 h, were processed for immunofluorescence analysis with antiserum (1:100) directed against SERPINE2, and revealed with an FITC-conjugated secondary antibody. DAPI was used to stain nuclei. Bar = 100 μm.
